# The causal effect of catastrophic health expenditure on poverty in Poland

**DOI:** 10.1007/s10198-023-01579-6

**Published:** 2023-03-10

**Authors:** Aleksandra Kolasa, Ewa Weychert

**Affiliations:** Faculty of Economic Sciences, University of Warsaw, Warsaw, Poland

**Keywords:** I32, I14, J14, Monetary poverty, Catastrophic health expenditure, Out-of-pocket medical expenses, Recursive probit models

## Abstract

**Introduction:**

Out-of-pocket medical expenses are a crucial source of health care financing in a number of countries. With the ongoing population aging, health care costs are likely to increase. Therefore, disentangling the relationship between health care spending and monetary poverty is becoming increasingly important. Although there is extensive literature on the impoverishment effect of out-of-pocket medical payments, it lacks empirical studies on a causal relationship between catastrophic health expenditure and poverty. In our paper, we try to fill this gap.

**Methods:**

We estimate recursive bivariate probit models using Polish Household Budget Survey data covering years from 2010 to 2013 and from 2016 to 2018. The model controls for a wide range of factors and endogeneity between poverty and catastrophic health expenditure.

**Results:**

We show that the causal relationship between catastrophic health expenditure and relative poverty is significant and positive across different methodological approaches. We find no empirical evidence that a one-time incidence of catastrophic health expenditure creates a poverty trap. We also show that using a poverty measure which treats out-of-pocket medical payments and luxury consumption as perfect substitutes can lead to an underestimation of poverty among the elderly.

**Conclusion:**

Out-of-pocket medical payments should probably receive more attention from policymakers than the official statistics suggest. A current challenge is to correctly identify and appropriately support those who are most affected by catastrophic health expenditure. More prospectively, a complex modernization of the Polish public health system is needed.

**Supplementary Information:**

The online version contains supplementary material available at 10.1007/s10198-023-01579-6.

## Introduction

Out-of-pocket medical (OPM) expenses are an important source of health care financing in many countries [52]. Therefore, their size and distribution can have a considerable impact on an individual’s well-being and the economy as a whole. In particular, households with large OPM payments can suffer from financial hardship and impoverishment [36]. With the ongoing aging of the worldwide population, health care costs are likely to continue increasing [18, 30]. Moreover, the recent COVID-19 pandemic exposed the vulnerability of the health care systems in many countries. Overall, a proper understanding of the relationship between OPM spending and poverty is becoming increasingly important.

This question is also particularly relevant in Poland, where the speed of population aging is one of the fastest in Europe [47]. As in the majority of European countries, Poland has free and universal healthcare. Yet, according to [35], total health spending in Poland is low and receives a relatively small share of public financing compared to other European countries. While the Polish health system is predominantly focused on hospital care, outpatient medicines account for the highest proportion of OPM payments. Since the probability of having pharmaceutical costs increases with age [40], elderly households in Poland are likely to be particularly affected by the burden of OPM payments.

Most studies that analyze OPM expenses in the context of poverty use catastrophic health expenditure (CHE) to identify households with an excessive financial burden due to health-related costs. Households experience CHE if their OPM spending is high in relation to their available resources. One line of research describes cross-country differences in CHE and investigates their sources (see, among others, [7, 44, 48, 50, 52–54], as well as a variety of country-specific WHO reports). These studies emphasize the importance of the design of the healthcare system, showing that higher OPM spending increases both the share of households affected by CHE and the overall poverty rate. Another common approaches are to examine the characteristics of households affected by CHE, and to study the determinants of CHE [1, 3, 9, 15, 24, 46, 58]. This line of the literature allows the extent of financial hardship to be quantified, typically finding that high OPM payments are particularly burdensome for the poor and elderly [41].

Assessing the overall impact of OPM payments on poverty is usually done by calculating the impoverishment effect. This measure shows the difference between actual and hypothetical poverty. In the hypothetical scenario, there are no OPM payments, and the available resources can finance basic household consumption. There is extensive literature that calculates the impoverishment effect for low- and middle-income countries, and a smaller body of research exists for high-income economies [4, 5, 8, 17, 29, 38]. In general, these studies find this effect to be substantial. Fewer studies look at the impact of CHE on transitions into and out of poverty. One example is a recent work by Kim and Kwon [23], who show that households experiencing CHE have lower chances of exiting from poverty to near-poverty.

In this paper, we contribute to the literature by investigating the relationship between OPM payments and monetary poverty using a somewhat different approach. Our goal is to quantify the average causal effect of large OPM spending (approximated by CHE) on the risk of poverty. To this end, we estimate a recursive bivariate probit model using Polish Household Budget Survey data which cover most of the 2010s. The model controls for a wide range of factors and endogeneity between poverty and CHE. Our modeling framework is related to the recent literature that addresses interdependence between social indicators with the help of multi-equation models [6, 21]. In particular, Maruotti [28] estimates determinants of CHE and impoverishment due to health spending for Italian households using a correlated random effects model. Since poverty and CHE measures are sensitive to methodological choices [13, 22, 55], we consider different thresholds and alternative approaches to the CHE measurement. To our knowledge, we are the first to examine the causal relation between CHE and poverty while correcting for endogeneity between CHE and poverty.

Our paper also adds to the debate on the appropriate poverty measure where OPM expenses are present [31, 49, 51]. Currently, the official measure of relative poverty, used by many statistical offices, including the Polish Central Statistical Office, is based on a household's total consumption expenditure. We move away from treating OPM payments and luxury consumption as perfect substitutes and offer a corrected estimates of poverty rate.

In addition, we present estimates of transition matrices for the incidence of CHE in Poland. To our knowledge, these are the first such estimates to have been calculated. We then compare the implied standard mobility indices of CHE and poverty dynamics.

## Definitions and concepts

### Relative poverty

Poverty is a multidimensional concept and refers to a state in which some basic human needs are not satisfied [2, 14]. In this study, we look at the monetary dimension of poverty. Individuals and households are at risk of monetary poverty if their available resources are below a certain threshold. This threshold can be set by reference to the costs of meeting basic needs, or to the standard of living of the whole community. In the case of latter, we talk about relative poverty. In many countries, official statistics on relative poverty are based on household consumption expenditure. In particular, such an approach is used by the Polish Central Statistical Office (CSO) to calculate poverty rates. According to this definition, a person is in relative poverty if the total consumption expenditure of his/her household are lower than 50% of the country average. Another common approach to assessing relative poverty is based on disposable income instead of consumption. In particular, this method is used in the official European statistics (EU-SILC), which define the poor as those whose disposable income is lower than some proportion of a country median.

It is well recognized that households necessarily incur certain critical costs, such as OPM and work-related expenses, and these costs vary with geographical factors and household composition [31]. Thus, instead of incorporating only the average of this spending in the poverty threshold, another option is to subtract it from the measure of household economic resources as, for example, is the practice of the US Census Bureau for the *Supplemental Poverty Measure* [43].

The cost of living depends on household size and composition. Therefore, poverty estimates typically use equivalised measures. More specifically, a household's income or consumption is divided by the appropriate equivalence scale, i.e., a weighted sum of all household members. In particular, the Polish CSO uses the original OECD equivalence scale, for which the weighting assigned to a household head is 1, the weighting used for children younger than 14 is 0.5, and all household members aged at 14 and over, except for the household head, are assigned a weighting of 0.7.

### Measuring relative poverty in the context of OPM expenses

Let us now consider how the standard measures of relative poverty respond to OPM payments. When equivalised income is used to indicate monetary poverty, health-related spending has no impact on the poverty status of a household or individual. By contrast, estimates of relative poverty based on total consumption are affected by the size of OPM expenses, but not always in a desirable way. Let us think of a household that is forced to reduce some of its basic consumption to meet some medical needs. At the same time, its total consumption, including OPM spending, is higher than it would be without a health shock. The thus measured poverty indicator for such a household would decrease.

A poverty rate calculated net of OPM payments would not have the above drawbacks. However, in this approach, all health-related expenses are treated as inevitable and necessary expenses. In real life, the size of OPM expenditure is not independent of an individual's financial situation. In particular, economic status is widely recognized as a risk factor for having unmet health care needs [36, 39].

Apart from the decision whether or not to subtract OPM expenses before calculating poverty status, one has to choose a proxy for household resources. The two most popular alternatives are consumption and income. In this context, it is worth noting that the possibility of experiencing idiosyncratic health shocks and resulting health-related spending have an effect on intertemporal household decisions [12]. More precisely, households can accumulate assets that help them smooth their consumption against health shocks. Using income as a poverty indicator would not account for this fact. Thus, for assessing the effect of large OPM expenses on relative poverty, consumption seems to be the more appropriate measure of household resources.

The data that we use in this study do not allow us to distinguish between essential and supplementary health spending, nor does it include sufficient information to appropriately approximate deferred health expenditure. For this reason, the poverty indicator that we use to assess the impact of CHE is based on total household consumption less the value of all health-related expenses paid directly by households.

In addition to OPM spending, there are other types of critical costs, such as work- and child-related payments, which might also be considered as necessary. These suffer from similar methodological and measurement issues as health-related expenses, which makes accounting for them a non-trivial task. As we want to keep our poverty indicator as close as possible to that used in official statistics, we have chosen not to subtract any more expenditures from the resource measure defined above.

We define the threshold that separates the poor from the not-poor as a proportion of average household consumption. Similar to the Polish CSO, the cutoff is set at 50%, and we use the original OECD equivalence scale. As a robustness check, we also perform the analysis for two different cutoffs: 45% and 65%.

All variables used in this study are at the household level, so a household is also the basic unit for our analysis. In official Polish CSO statistics, poverty is determined at the household level, but poverty rates are calculated per capita, i.e. they show the proportion of the population living in poverty. For this reason, the aggregate statistics presented in this paper and the corresponding official estimates might differ slightly.

### Catastrophic health expenditure

OPM payments include all spending on health-related goods and services borne directly by households. For households with different socio-economic status, problematic levels of such payments can be different. Hence, in the literature, it is common to focus on the individuals whose OPM expenses constitute a large fraction of their resources. If this fraction exceeds a certain threshold, an incidence of catastrophic health expenditure (CHE) occurs. There are two main ways to measure CHE. The first one is called the *budget share* approach, and OPM payments are expressed as a share of total household consumption or income. Another method is to examine a household's capacity to pay for health-related goods and services. In this case, we look at OPM payments in relation to a household's remaining consumption, which is total consumption less spending on basic needs. The most common proxies for the costs of basic needs are actual household food consumption (actual food spending approach) and a standard amount of food spending (the normative food spending approach, see [55]. In recent WHO reports, spending on housing and utilities is also included in the costs of basic needs. A detailed discussion on the advantages and drawbacks of using different CHE measures can be found in Box 2 in a most recent WHO report [52] on financial protection. In general, in the *capacity to pay* approach, CHE is more concentrated among poor households compared to the *budget share* method [11].

There is no consensus on a single threshold that can be applied to identify an incidence of CHE. The majority of studies that focus on European countries use thresholds between 10 and 25% in the case of the *budget share* approach, and thresholds of around 40% when using the *capacity to pay* method [55].

To evaluate the sensitivity to different CHE measures and thresholds, the results presented in the paper will be based on two standard approaches for identifying an incidence of CHE, which are the *budget share* approach with thresholds set at 10%, 15% 20%, and 25%, and the *normative spending* approach with thresholds at 25%, 30%, 35%, and 40%. For the latter, we approximate the cost of basic needs with the minimum of the following two values: actual household spending on food, housing, and utilities, and an average of such spending calculated for households between the 25th and 35th quantile in the consumption distribution. We also adopted two alternative measures of CHE: an *actual food spending* approach and a *normative food spending* approach. As the estimates obtained with these two approaches did not change any of the main findings of this paper, they are not presented here, but we can make them available on request.

## Data

In this study, we use household-level data from the Polish Household Budget Survey (HBS). The HBS provides a rich database on the income, consumption, and a variety of socioeconomic characteristics of Polish households. In particular, it is the only database with such precise and detailed information on household spending on health-related goods and services. The HBS data are used for calculating various official estimates, including consumption-based poverty.

Every year, the survey is conducted on a sample of approximately 37 thousand Polish households. Each household is interviewed exactly twice in the two subsequent years. The detailed data on household expenditure and income are taken from one randomly selected month. In the next section, we show selected statistics based on data just from one year—2018. In Sects. "Mobility indices" and "The causal effect of CHE on poverty", we present analysis based on data from the following five panels: 2010–2011, 2011–2012, 2012–2013, 2016–2017, and 2017–2018. We excluded households in which the household head did not remain the same for both interviews. That gives us 74,186 observations in total. The number of observations for each panel is listed in the Supplementary Appendix.

The Polish HBS database provides survey weights for cross-sectional data collected in a given year. When calculating the transition matrices, each household takes the weight assigned during its first interview. However, our results remain qualitatively unchanged if we use unweighted data.

## Relative poverty, OPM payments, and CHE in Poland

### Relative poverty

Relative poverty in Poland is moderate in comparison to other OECD countries [34]. The introduction of the universal child benefit *500 Plus* in 2016 was supposed to decrease it even further (see preliminary predictions by [20], and [10]). However, according to the Polish CSO, the share of the population at risk of relative poverty dropped only from 13.9% in 2016 to 13.0% in 2019, despite favorable economic conditions.

**Fig. 1:**
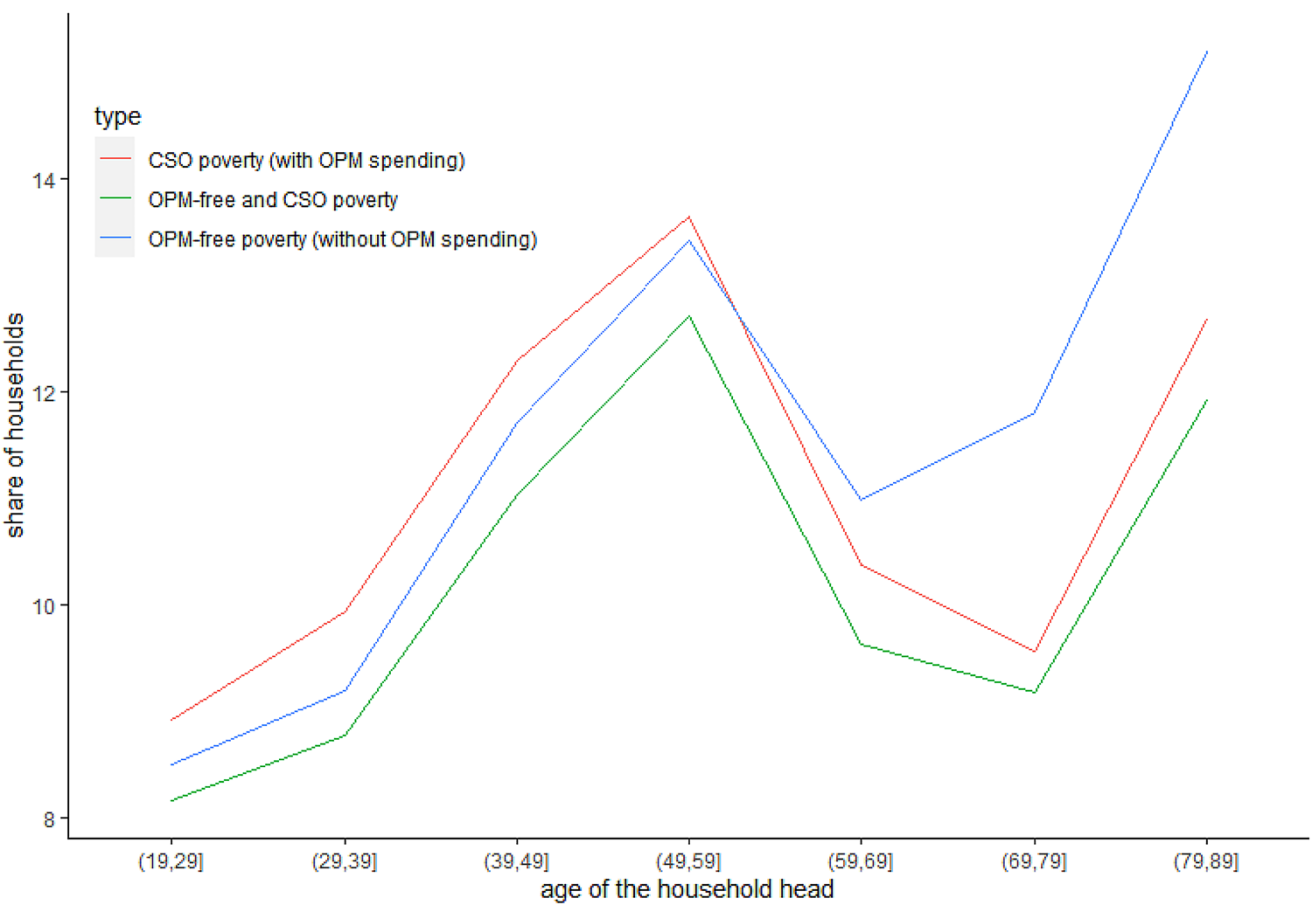
Relative poverty rates over age. Notes: Authors' estimates based on 2018 Polish HBS data; red line: relative poverty rate calculated on the basis of total consumption (CSO poverty rate); blue line: relative poverty rate calculated on the basis of total consumption minus OPM expenses (OPM-free poverty rate)

In Fig. 1, we show how the relative poverty rates vary with the age of the household head. The estimates are based on the Polish Household Budget Survey (HBS) data from 2018. The red line represents consumption-based relative poverty rates calculated using the measurement approach adopted by the Polish CSO. We refer to poverty thus measured as CSO poverty. The blue line shows relative poverty rates based on household consumption without OPM expenses, which we refer to as OPM-free poverty. An examination of Fig. 1 reveals that the share of households in relative poverty increases with household age up to the age of 50, and then declines. This trend is reversed again for households aged between 60 and 80. In general, the two relative poverty measures overlap, especially for younger households. However, the estimates of the OPM-free poverty rate indicate a 2.4 percentage point higher share of poverty among elderly households. Moreover, the CSO poverty rate of elderly households (70 years old or more) is lower than that of the population as a whole, which is not the case for the OPM-free poverty rate (Table 1). Other studies confirm the elderly's vulnerability to poverty, and thus are more in line with the OPM-free poverty rate. In particular, Eurostat (based on EU-SILC data) reports that the risk of poverty or social exclusion for individuals aged 60 or more is 1 percentage point higher than for the entire population.

**Table 1:** Poverty rate by age groups and poverty indicators

Age of household head	CSO poverty rate	OPM-free poverty rate
less than 70	11.30%	11.10%
70 and more	10.60%	13.00%

Authors' estimates based on 2018 Polish HBS data; CSO poverty: relative poverty calculated on the basis of total consumption; OPM-free poverty: relative poverty calculated on the basis of total consumption minus OPM expenses

### OPM payments

According to the [35], Poland has one of the lowest total healthcare spending levels (around 6.5% of GDP in 2017) and one of the highest out-of-pocket pharmaceutical expenditures among European countries. The share of OPM payments in total health expenditure in Poland has not changed much over the last ten years, accounting for approximately one-fifth of all health expenditure (the World Bank database). Pharmaceutics account for most OPM spending (around 3/5), followed by dental care (1/6) and outpatient medical care (1/7).

OPM expenses make up about 5% of total household consumption (2018 Polish HBS). Elderly households (with household heads aged 70 or over) spend slightly less than 1/10 of their consumption on health goods and services, more than twice as much as other households. Furthermore, Tambor and Pavlova [46] report that in Poland senior citizens, the chronically ill, and the disabled have a higher probability of not being able to afford to purchase prescribed drugs than the rest of the population.

### Catastrophic health expenditure

Tambor and Pavlova [46] look closely at the magnitude and distribution of CHE in Poland. According to their findings, the incidence of CHE in Poland does not stand out from other European countries. Between 2005 and 2014, it followed a moderate downward trend, with a more profound decrease for households in the middle three consumption quintiles. According to Tambor and Pavlova [46], CHE is highly concentrated among the poorest. Łuczak and García-Gómez [25] and Zawada et al. [57] also confirm this finding for different threshold levels. Furthermore, Łuczak and García-Gómez [25] point to a significant impoverishment effect of CHE in Poland. According to their estimates, the relative poverty rate in 2009 was 4.9 percentage points higher due to out-of-pocket pharmaceutical payments. In general, OPM payments have a larger impoverishment effect for seniors and the chronically ill.

**Fig. 2:**
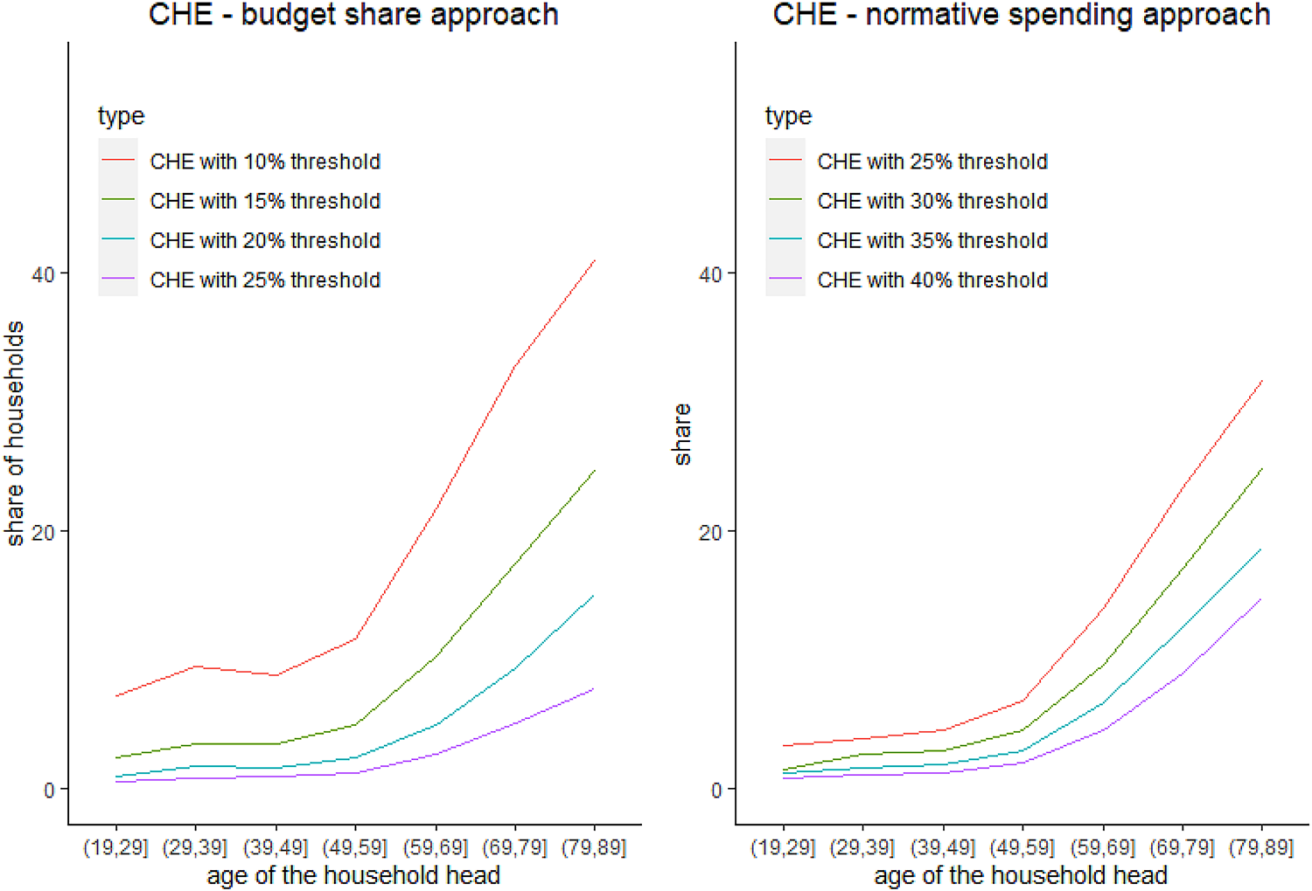
Shares of households with CHE according to age. Notes: Authors’ estimates based on 2018 Polish HBS data; CHE denotes catastrophic health expenditure

The share of households with CHE varies greatly with measurement and threshold choices. Indeed, according to our estimates calculated using the 2018 Polish HBS data, the incidence of CHE was between 2 and 17% for the budget share approach when thresholds were varied incrementally from 10 to 25%. For the normative spending approach and incremental thresholds on a scale from 25 to 40%, the share of households with CHE ranged between 4 and 10% (see Fig. 2). However, regardless of methodological choices, the incidence of CHE grows considerably with the age of the household head from 60 years old and onwards. The share of households with CHE among households with household heads aged 65 or more is between 3 and 5 times higher than in the rest of the population.

### Relative poverty and CHE

**Fig. 3:**
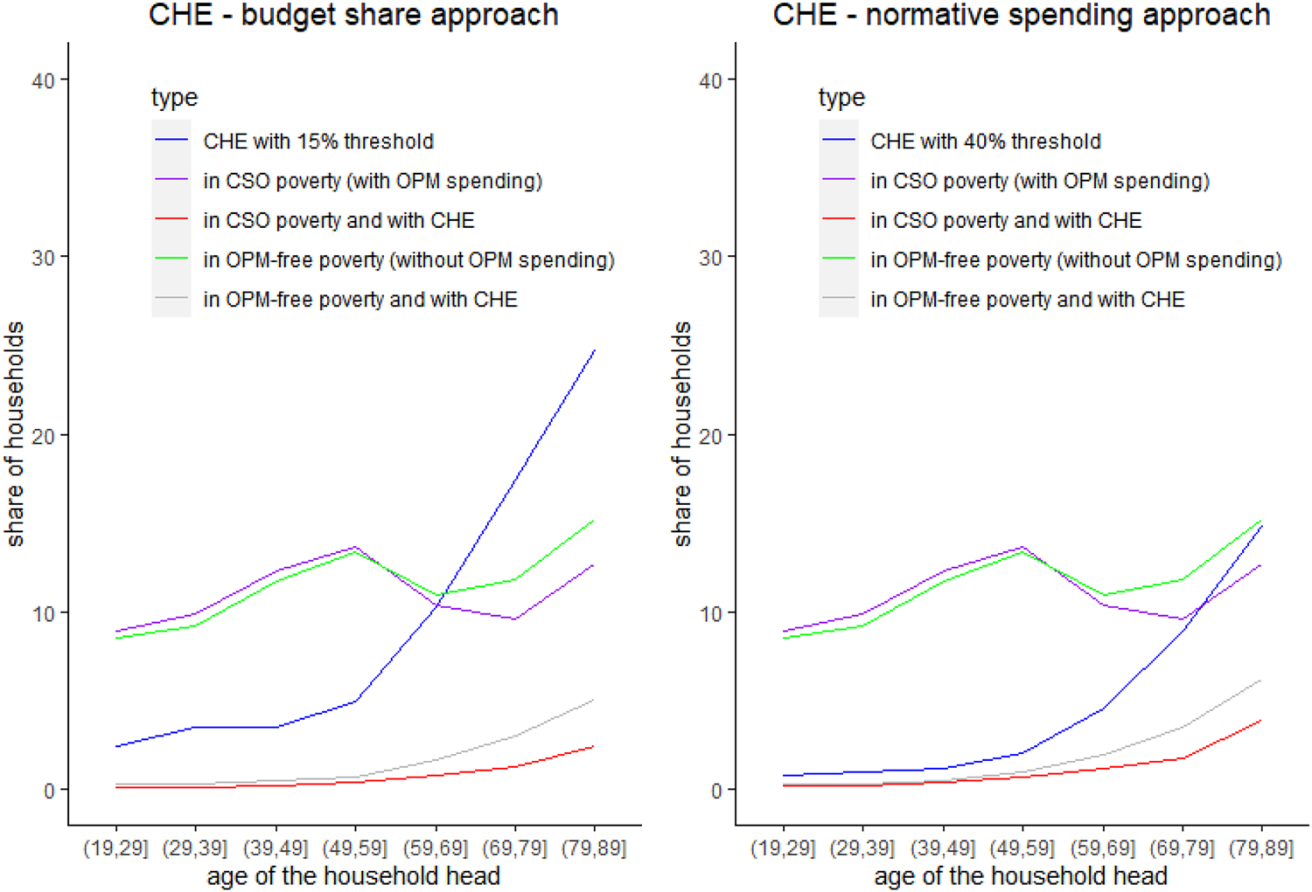
Relative poverty and households with CHE according to age. Notes: Authors’ estimates based on 2018 Polish HBS data; CHE denotes catastrophic health expenditure and OPM out-of-pocket medical payments

Let us finally look at the interactions between relative poverty and CHE in relation to age, using the 2018 Polish HBS data. We focus on two CHE measures: the first one calculated with the budget share approach using a threshold of 15% (left panel of Fig. 3), and the second one based on the normative spending approach for a threshold of 40% (left panel of Fig. 3). The gray line in Fig. 3 shows the share of households that have CHE and are in OPM-free poverty, while the red line represents those experiencing CHE and CSO poverty.

First, Fig. 3 indicates a higher share of households with CHE among those in OPM-free poverty compared to those in CSO poverty. In particular, around 13% of households in OPM-free poverty have CHE (calculated using the normative spending approach), but the incidence of CHE is only 8% for those in CSO poverty (Table 2). For the budget share approach, these numbers are 11% and 5% respectively. Regardless of the measure, the majority of poor households do not have CHE.

**Table 2:** The intersection of poverty and CHE

	Households in OPM-free poverty	Households in CSO poverty
Share of households with CHE (normative spending approach, threshold = 40%)	13%	8%
Share of households with CHE (budget share approach, threshold = 15%)	11%	5%

	Households with CHE (normative spending approach, threshold = 40%)	Households with CHE (budget share approach, threshold = 15%)
Share of households in OPM-free poverty	42%	16%

Authors’ estimates based on 2018 Polish HBS data; CSO poverty: relative poverty calculated on the basis of total consumption; OPM-free poverty: relative poverty calculated on the basis of total consumption minus OPM expenses

Second, in line with the related literature, our estimates of CHE indicate that using the normative spending approach gives an incidence of CHE which is more concentrated among the poor. Indeed, 42% of households with CHE based on the normative spending approach are in OPM-free poverty, and only 16% of those with CHE calculated using the budget share approach are identified as (OPM-free) poor.

Finally, as the risk of having CHE increases for elderly households, so do the shares of households that are both in poverty and have CHE.

## Mobility indices

Having documented the correlation between relative poverty and CHE using cross-sectional data, we now focus on their dynamics. We evaluate the differences in poverty mobility and the mobility of the incidence of CHE. We also check whether having CHE might change the patterns of poverty persistence.

To this end, we use transition matrices, calculated for different groups and measurement approaches (Tables 3, 4, 5). A transition matrix shows the proportions of households that are poor/non-poor (or with/without CHE) in a particular year, broken down by their poverty (or CHE) status in a previous year. In addition, the Supplementary Appendix contains one-dimensional statistics of the immobility ratio and the Shorrocks [42] mobility index that helps to summarize the overall degree of mobility and statistical significance of the results.

**Table 3:** Poverty transition matrices

	CSO indicator			OPM-free indicator	
	Poor ($t$)	Not-poor ($t$)		Poor ($t$)	Not-poor ($t$)
Poor ($t-1$)	53.3	46.7	Poor ($t-1$)	52.4	47.6
Not poor ($t-1$)	6.3	93.7	Not poor ($t-1$)	6.4	93.6
Shares at time $t$	12.1	87.9	Shares at time $t$	12.3	87.7

Notes: Authors’ estimates based on the Polish HBS data, number of observations is 74,186; CSO indicator: relative poverty calculated on the basis of total consumption; OPM-free indicator: relative poverty calculated on the basis of total consumption minus OPM expenses

**Table 4:** OPM-free poverty transition matrices of households with CHE

Households with CHE at time *t*-1					
	Budget share approach (threshold = 15%)		Normative spending approach (threshold = 40%)		
	Poor (*t*)	Not-poor (*t*)		Poor (*t*)	Not-poor (*t*)
Poor ($t-1$)	48.1	51.9	Poor ($t-1$)	50.7	49.3
Not poor ($t-1$)	6.1	93.9	Not poor ($t-1$)	8.8	91.2
Shares at time $t$	14.2	85.8	Shares at time $t$	23.7	76.3
Obs. no.: 6390			obs. no.: 4063		

Households with CHE at time *t*					
	Budget share approach (threshold = 15%)		Normative spending approach (threshold = 40%)		
	Poor (*t*)	Not-poor (*t*)		Poor (*t*)	Not-poor (*t*)
Poor ($t-1$)	61.9	38.1	Poor ($t-1$)	73.5	26.5
Not poor ($t-1$)	11.1	88.9	Not poor ($t-1$)	21.6	78.4
Shares at time $t$	17.7	82.3	Shares at time $t$	34.4	65.6
Obs. no.: 6168			Obs. no: 3935

**Table 5:** Transition matrices for the incidence of CHE

Budget share approach					
	Threshold = 15%			Threshold = 25%	
	With CHE ($t$)	Without CHE ($t$)		With CHE ($t$)	Without CHE ($t$)
With CHE ($t-1$)	32.9	67.1	With CHE ($t-1$)	18.6	81.4
Without CHE ($t-1$)	6.0	94.0	Without CHE ($t-1$)	1.8	98.2
Shares at time $t$	8.3	91.7	Shares at time $t$	2.2	97.8

Normative spending approach					
	threshold = 25%			Threshold = 40%	
	With CHE ($t$)	Without CHE ($t$)		With CHE ($t$)	Without CHE ($t$)
With CHE ($t-1$)	40.7	59.3	With CHE ($t-1$)	30.0	70.0
Without CHE ($t-1$)	8.9	91.1	Without CHE ($t-1$)	3.6	96.4
Shares at time $t$	13.3	86.7	Shares at time $t$	4.9	95.1

Authors’ estimates based on the Polish HBS data, number of observations is 74,186

Table 3 shows the poverty transition matrices calculated for the two indicators of relative poverty, OPM-free poverty and CSO poverty. For both poverty measures, we observe a similar degree of mobility. The share of households remaining in relative poverty for at least two years is between 52 and 53%, while the share of those that escape poverty every year slightly exceeds 6%.

Next, we look at poverty transitions of households with CHE (Table 4). We calculate CHE here using the two most common methodologies, i.e., the normative spending approach with a threshold of 40%, and the budget share approach with a threshold of 15%. Once again, our estimates confirm that CHE and poverty are positively correlated. If we take a household that has CHE at time $t$, its probability of staying in poverty at time $t$ is 10–20 percentage points higher than that of the entire population. There are significant differences between the results depending on the CHE measure used. In general, households which are determined to have CHE on the basis of the normative spending approach have higher probabilities of remaining in/falling into poverty than households determined to have CHE under the budget share approach.

However, according to the transition matrices, there is no evidence that an incidence of CHE increases the risk of poverty in the next period (Table 4). On the contrary, the mobility indices suggest a slightly higher poverty mobility for households with CHE at time $t-1$ compared to the entire population. This result might suggest that having CHE does not create a poverty trap. Indeed, after the initial shock of CHE, households might use their savings or other resources to finance medical needs, and thus be able to return to their previous consumption level.

However, this might not be the whole picture. Consider individuals who do not have a sufficient financial buffer. They might be incapable of bearing high medical costs for an extended period, and, as a result, could end up with unmet health needs. Unfortunately, our data do not allow us to quantitatively verify these hypotheses.

The incidence of CHE has significantly lower persistence than relative poverty, for all considered thresholds and measurement approaches (see Table 5 and the Supplementary Appendix for more thresholds). Intuitively, the higher the threshold, the greater the mobility. For the two most common CHE measures, only less than one-third of households with CHE at time $t-1$ experience CHE at time $t$. As a robustness check, we also calculated the mobility indices using single two-year panels. The results, available on request, confirm our findings described above.

## The causal effect of CHE on poverty

### Econometric model

We have shown so far that households experiencing CHE are more likely to fall into poverty and remain in it. However, this fact alone does not imply causality. Indeed, there might be factors, such as age or disability, which simultaneously influence both poverty and the incidence of CHE. If some of these factors are unobservable, endogeneity arises.

We address this problem using joint bivariate regression models. Our modeling framework consists of two simultaneous probit equations: one for poverty and one for CHE. The error terms of these equations can be correlated. More specifically, we estimate the following model:

1 $${POV}_{s}^{*}={\phi }_{1}{POV}_{s,-1}+{\phi }_{2}{CHE}_{s}+{\phi }_{3}{CHE}_{s,-1}+\beta {X}_{s}+{\varepsilon }_{s}$$

2 $${CHE}_{s}^{*}={\rho }_{1}{POV}_{s,-1}+{\rho }_{2}{CHE}_{s,-1}+\Upsilon {X}_{s}+{\tau }_{s},$$

$$cov(\varepsilon_{s} , \tau_{s} ) = \theta ,{ } cov(\varepsilon_{s} , \varepsilon_{s - r} ) = 0,{ } cov(\tau_{s} , \tau_{s - r} ) = 0,{{ for\, r }} \ne 0$$

$$POV_{s} = {\text{I}}\left( {POV_{s}^{*} > 0} \right),{ } CHE_{s} = I\left( {CHE_{s}^{*} > 0} \right),$$

where ${POV}_{s}$ and ${POV}_{s,-1}$ are binary variables indicating the current and previous poverty status of a household $s$, respectively. ${CHE}_{s}$ equals one if a household $s$ currently has CHE, while ${CHE}_{s,-1}$ refers to an incidence of CHE in the previous year. $X$ is a vector of other explanatory (exogenous) variables, ${\phi }_{1}$, ${\phi }_{2}$, ${\phi }_{3}$, ${\rho }_{1}$, ${\rho }_{2}$, β, γ are parameters and ϵ, τ are error terms. We refer to parameters ${\phi }_{1}$ and ${\rho }_{2}$ as reflecting state dependence of poverty and of CHE, respectively.

We estimate the model using R package GJRM [26] and Gaussian copula. Apart from poverty and the incidence of CHE, the explanatory variables include: the number of children, working and non-working adults in a household, binary variables indicating the age, sex, education level, and relationship status of the household head (HH), the presence of disabled household members, the predominant source of income of household members, the type of area in which a household lives, and dummies for years. The definitions and descriptive statistics of all variables are presented the Supplementary Appendix. As we discussed in Sect. "Definitions and Concepts", to properly capture the effect of CHE on poverty, OPM payments should not be included in household resources. Thus, from now on, our only poverty indicator is OPM-free poverty.

### Results

With the gradient test [27], the hypothesis of no endogeneity was rejected at a significance level of 5% for all considered CHE thresholds in the case of the budget share approach and for the 25% threshold in the case of the normative spending approach. This confirms the need to use joint bivariate regression models in our analysis.

### State dependence and feedback effect

In Table 6, we present the average marginal effects (AME) of poverty and CHE based on the model described in the previous subsection. The table contains the estimates of state dependence, as well as the feedback effects from CHE to poverty and vice versa. The *poverty equation* refers to Eq. 1, while the *CHE equation* refers to Eq. 2. The AME of lagged poverty on current poverty captures poverty state dependence. Similarly, state dependence of CHE is approximated by the AME of an incidence of CHE in the previous year on a current incidence of CHE.

**Table 6:** State dependence and feedback effects, poverty and CHE

Budget share approach								
th.	10%		15%		20%		25%	
	Poverty equation							
	AME	s.e	AME	s.e	AME	s.e	AME	s.e
POV_− 1_	0.325 ***	0.120	0.323 ***	0.117	0.319 ***	0.114	0.319 ***	0.113
CHE	0.142 ***	0.078	0.168 ***	0.083	0.277 ***	0.107	0.346 ***	0.116
CHE_− 1_	− 0.035 ***	0.028	− 0.029 ***	0.024	− 0.034 ***	0.029	− 0.034 ***	0.029
	CHE equation							
	AME	s.e	AME	s.e	AME	s.e	AME	s.e
POV_− 1_	− 0.022 ***	0.011	− 0.006 *	0.004	0.000	0.000	0.002	0.002
CHE_− 1_	0.195 ***	0.055	0.150 ***	0.066	0.112 ***	0.064	0.080 ***	0.052
Normative spending approach								
th.	25%		30%		35%		40%	
	Poverty equation							
	AME	s.e	AME	s.e	AME	s.e	AME	s.e
POV_− 1_	0.303 ***	0.12	0.309 ***	0.118	0.311 ***	0.116	0.311 ***	0.115
CHE	0.211 ***	0.100	0.171 ***	0.087	0.164 ***	0.083	0.182 ***	0.087
CHE_− 1_	− 0.02 ***	0.017	− 0.015 **	0.012	− 0.013 *	0.01	− 0.013 *	0.011
	CHE equation							
	AME	s.e	AME	s.e	AME	s.e	AME	s.e
POV_− 1_	0.030 ***	0.019	0.023 ***	0.017	0.025 ***	0.02	0.025 ***	0.022
CHE_− 1_	0.158 ***	0.068	0.155 ***	0.074	0.138 ***	0.077	0.117 ***	0.075

Authors’ estimates based on the recursive bivariate probit models and the Polish HBS panel; AME expresses the average marginal effect of the change from 0 to 1; ***, **, *, and. denote parameter significance at the 0.1%, 1%, 5% and 10% levels, respectively

The estimates point to a high degree of state dependence of relative poverty. Poverty in the previous year increases its risk in the current year by more than 30 percentage points, and this result is robust across all specifications. This finding indicates that Poland has a much higher level of poverty state dependence compared to the European average, but similar to countries such as Greece or Turkey [19, 33, 56]. Our result is in line with Ayllón and Gábos [6], who also find that poverty in Poland is strongly affected by state dependence. A high degree of poverty state dependence might suggest that the experience of poverty depreciates human capital and decreases motivation and incentives to change unfavorable conditions. Thus, this finding stresses the importance of public measures not only to alleviate already existing poverty, but also to prevent households from falling into poverty in the first place.

The total effect of CHE on poverty is positive and significant for all specifications. A new incidence of CHE calculated with the normative spending approach increases the risk of poverty by 22–27 percentage points. In the case of the budget share approach, this increase can reach even 34 percentage points. However, for the two most common thresholds, i.e., 10% and 15%, having CHE increases the poverty risk by 14 and 17 percentage points, respectively.

We do not observe higher poverty risk for households with CHE in the previous year. This means that having an incidence of CHE does not negatively impact a household's future experience of poverty. On the contrary, an incidence of CHE slightly decreases the risk of poverty in the following year. While this effect is small in magnitude and insignificant for the normative spending approach with high threshold levels, it might be still worth discussing why a household with CHE in the two subsequent years would have a slightly lower risk of poverty than a similar household with CHE only in the current year. The economic explanation of this finding might be as follows. After the initial shock of a new incidence of CHE, households might finance their OPM expenditures by reducing their current consumption. But once they realize that they will need to bear health-related costs for longer, some of them might seek other sources of financing for OPM payments, such as additional income, the sale of some assets, or borrowing from family. As a result, their consumption might return to normal levels, or at least be at a higher level than in the previous year.

Our results confirm the existence of state dependence of the incidence of CHE. Having CHE in one year translates into a 6.6–19.5 percentage point higher risk of experiencing CHE also in the following year. The estimated effect varies with the approach and thresholds used, but poverty exhibits a much higher degree of state dependence than the incidence of CHE for all specifications.

The results for the impact of lagged poverty on a current incidence of CHE are mixed. For the normative spending approach, being poor in one year increases the risk of having CHE in the following year by around 1 percentage point. For the budget share approach, the effect of lagged poverty on CHE is insignificant for all but one of the thresholds.

### Risk ratios and odds ratios

In Table 7, we show the poverty risk ratios and odds ratios for a new incidence of CHE. For the normative spending approach and its most common threshold (i.e., 40%), the probability of relative poverty is 2.6 times higher for a household with a new incidence of CHE compared to a similar one without CHE. For this case, the odds ratio is 3.3. If we take the budget share approach with its most common threshold (15%), the estimated effects are of very similar magnitude. In general, the risk ratios are between 2.3 and 3.8, and the odds ratios between 2.8 and 6.3 for all considered specifications.

**Table 7:** The causal impact of a new incidence of CHE on relative poverty

Budget share approach				
threshold	10%	15%	20%	25%
Risk ratio	2.31 [1.82, 2.93]	2.45 [1.81, 3.16]	3.33 [2.61, 4.31]	3.84 [2.71, 4.98]
Odds ratio	2.75 [2.10, 3.80]	3.02 [2.00, 4.41]	4.85 [3.14, 7.69]	6.34 [3.82, 10.34]

Normative spending approach				
Threshold	25%	30%	35%	40%
Risk ratio	3.03 [2.49, 3.62]	2.56 [2, 3.26]	2.45 [1.82, 3.04]	2.58 [1.88, 3.38]
Odds ratio	3.96 [2.72, 5.37]	3.17 [2.47, 4.31]	3.00 [2.14, 4.25]	3.25 [2.28, 4.83]

Authors' estimates based on the recursive bivariate models and the Polish HBS panel; 95% confidence intervals are based on posterior simulations

### Control variables

In Table 8, we present the average marginal effects (AME) of the control variables from the poverty equation for normative spending approach (see Eq. 1, estimates for the budget share approach are very similar and can be found in the Supplementary Appendix). The estimates are robust across different specifications and have intuitive signs. The poverty risk is highest for middle-aged and young households. Being in a relationship reduces the risk of poverty by around 3 percentage points. Each child increases the risk of poverty by 3.2–3.4 percentage points, and each non-working adult in the household by 3.7–3.8 percentage points. As expected, the risk of poverty is negatively associated with the number of working adults in a household, each reducing the risk of poverty by slightly less than 2 percentage points. Similar to previous studies, we also estimate a significant reduction in the risk of poverty (by more than 7 percentage points) for more educated households, i.e., those where household heads have an academic degree. These results confirm the well-known empirical finding that education and employment are correlated with a higher income status.

**Table 8:** Poverty equation, AME of control variables

	Normative spending approach							
Threshold	25%		30%		35%		40%	
	AME	s.e	AME	s.e	AME	s.e	AME	s.e
HH age < 35 and > 24	0.021	0.017	0.024*	0.018	0.025*	0.018	0.025*	0.018
HH age < 45 and > 34	0.019	0.015	0.02	0.016	0.021	0.016	0.021	0.016
HH age < 55 and > 44	0.025*	0.019	0.027*	0.020	0.028*	0.021	0.028*	0.021
HH age < 65 and > 54	0.014	0.011	0.017	0.013	0.019	0.014	0.019	0.014
HH age < 75 and > 64	− 0.010	0.008	− 0.005	0.004	− 0.001	0.001	0.001	0.001
HH age > 74	− 0.002	0.002	0.008	0.006	0.013	0.01	0.014	0.011
HH is a male	0.000	0.000	− 0.001	0.001	− 0.001	0.001	− 0.002	0.001
HH is in a relationship	− 0.028***	0.022	− 0.03***	0.023	− 0.029***	0.022	− 0.029***	0.022
HH has academic degree	− 0.071***	0.066	− 0.072***	0.065	− 0.072***	0.064	− 0.072***	0.064
Income from farming	0.017***	0.013	0.018***	0.013	0.019***	0.014	0.018***	0.014
Income from self-emp	− 0.017***	0.015	− 0.018***	0.015	− 0.019***	0.015	− 0.019***	0.016
Lives in a town	− 0.034***	0.03	− 0.035***	0.030	− 0.035***	0.03	− 0.035***	0.030
Lives in a village	0.034***	0.027	0.035***	0.027	0.035***	0.026	0.035***	0.026
Disabled in household	− 0.001	0.001	0.000	0.000	0.001	0.001	0.002	0.001
No. of working	− 0.016***	0.116	− 0.017***	0.117	− 0.017***	0.12	− 0.018***	0.121
No. of not working	0.037***	0.16	0.037***	0.157	0.037***	0.156	0.038***	0.156
No. of children	0.033***	0.155	0.034***	0.153	0.033***	0.151	0.033***	0.150

Authors' estimates based on the recursive bivariate probit models and the Polish HBS panel; In the case of binary variables, AME expresses the average marginal effect of the change from 0 to 1, while, in the case of the remaining variables, it is a one-unit change, where a unit equals 1; ***, **, *, and. denote parameter significance at the 0.1%, 1%, 5% and 10% levels, respectively; Dummies for years and regions are included

Living in a town reduces the risk of poverty by around 3.6 percentage points, while living in a village increases it by approximately the same magnitude. Being dependent on farming for income increases the risk of poverty by around 2 percentage points, while self-employment works in the opposite direction, lowering the risk of poverty by around 2 percentage points. Finally, the presence of a disabled person in a household and having a male household head turn out to be statistically insignificant.

As a robustness check, we also estimate the models for different poverty cutoffs (i.e., 45% and 55% of mean consumption). The estimates obtained do not change our main findings (see the Supplementary Appendix).

## Concluding remarks

Using Polish micro-level data, we have shown that the causal effect of having CHE on the current risk of relative poverty is significant and positive across different methodological approaches. The estimated probability of relative poverty is 2 to 4 times higher for a household with a "new" incidence of CHE (i.e., it had no CHE in the previous year) compared to a household without CHE either currently or in the previous year, and the odds ratios are slightly less than 3 for the most common CHE thresholds. However, we have found no empirical evidence that a one-time incidence of CHE causes a poverty trap. Relative poverty exhibits a significantly higher degree of state dependence than CHE. Thus, it is much easier for a household to escape CHE than to escape poverty. Moreover, we show that the Polish official poverty statistics might not fully capture people impoverished due to CHE. As the elderly have the highest incidence of CHE, the share of households in relative poverty among those aged 70 and over might be underestimated by up to 2.5 percentage points by the Polish CSO.

Embodied in the Polish constitution, statutory health coverage in Poland is universal, with more than 90% of the population entitled to public health benefits. At the same time, as demonstrated in this paper, catastrophic health-related spending is a considerable risk factor for household monetary poverty. The above finding is striking and might indicate major service delivery issues in the public health sector. Indeed, the Polish health system struggles with personnel shortage, having one of the lowest numbers of doctors and nurses per person in Europe [45]. Public health care in Poland is also heavily focused on hospital care at the expense of outpatient and long-term care. The above is reflected by long waiting lines, especially for specialists or elective (non-urgent) surgeries, and limited public financing of dental care and outpatient medicines [37, 45].

The reforms implemented in the last decade, which include the creation of the hospital network and the reforms in primary care, address some of these problems. The former aims to improve the hospital payment system and coordination of services, while the latter seeks to enhance access to primary care. In general, these policies go in the right direction, but critics point to their ad-hoc character and the lack of a broader long-term strategy [16, 32]. The government's commitment to spend 6% of GDP on public expenditures on health, starting in 2023, offers scope for further improvement of the health service. However, the possible positive effects are not yet reflected in our results. The health system modernization carried out properly might grant broader access to public financing and increase the quality of service. Consequently, it might also decrease the incidence of CHE and make households financially less vulnerable to health shocks.

With the shortcomings of the Polish public health care system, private financing helps meet health needs and ensure the quality of service. Indeed, private spending accounts for around 28% of all health-related expenses. Moreover, approximately 2.3 and 3 million Poles have private health insurance cover and medical subscriptions, respectively [45]. However, relying on private resources is not always an option, especially for households with low income. They might instead postpone health-related spending and have unmet basic health care needs. According to Eurostat 2019, around 8.5% of Poles over 16 years old report unmet needs for medical examination or treatment, more than twice the European Union average. We do not see OPM payments in such cases, even if they are considered urgent. Thus, when one focuses on essential health-related expenditures instead of the actual ones, the impact of CHE on poverty would be even larger than that estimated in the paper.

Given our results and these additional arguments, OPM payments should probably receive more attention from policymakers than the official statistics suggest. A current challenge is to correctly identify and appropriately support those who are most affected by CHE. More prospectively, a complex modernization of the Polish public health system is needed.

## Supplementary Information

Below is the link to the electronic supplementary material. 

Supplementary file1 (DOCX 47 KB)

## Data Availability

The data presented in this article and all codes are available upon request. The study uses the Polish Household Budget Survey data, which are not publicly available but can be requested from the Polish Central Statistical Office. Due to the data access policy, we are unable to directly share the raw HBS data. However, interested researchers may contact the Polish CSO to request access to the data.

## References

[1] Ahmed S, Szabo S, Nilsen K (2018). Catastrophic healthcare expenditure and impoverishment in tropical deltas: evidence from the Mekong Delta region. Int. J. Equity Health.

[2] Alcock P (1997). Understanding poverty.

[3] Aregbeshola BS, Khan SM (2018). Determinants of catastrophic health expenditure in Nigeria. Eur. J Health Econ..

[4] Aregbeshola BS, Khan SM (2018). Out-of-pocket payments, catastrophic health expenditure and poverty among households in Nigeria 2010. Int. J. Health Policy Manag..

[5] Arsenijevic J, Pavlova M, Groot W (2013). Measuring the catastrophic and impoverishing effect of household health care spending in Serbia. Soc. Sci. Med..

[6] Ayllón S, Gábos A (2017). The interrelationships between the Europe 2020 poverty and social exclusion indicators. Soc. Indic. Res..

[7] Baird K (2016). High out-of-pocket medical spending among the poor and elderly in nine developed countries. Health Serv. Res..

[8] Bredenkamp C, Mendola M, Gragnolati M (2011). Catastrophic and impoverishing effects of health expenditure: new evidence from the Western Balkans. Health Policy Plan..

[9] Brown S, Hole AR, Kilic D (2014). Out-of-pocket health care expenditure in Turkey: analysis of the 2003–2008 household budget surveys. Econ. Model..

[10] Brzeziński M, Najsztub M (2017). The impact of family 500+ programme on household incomes, poverty and inequality. Polityka Społeczna.

[11] Cylus J, Thomson S, Evetovits T (2018). Catastrophic health spending in Europe: equity and policy implications of different calculation methods. Bull. World Health Organ..

[12] De Nardi M, French E, Jones JB (2010). Why do the elderly save? The role of medical expenses. J. Polit. Econ..

[13] Deaton A (1997). The analysis of household surveys: a microeconometric approach to development policy.

[14] Deaton A (2006). Measuring poverty. Understanding poverty.

[15] Dhak B (2015). Demographic change and catastrophic health expenditure in India. Soc. Indic. Res..

[16] Dubas-Jakóbczyk K, Kowalska-Bobko I, Sowada C (2019). The 2017 reform of the hospital sector in Poland – the challenge of consistent design. Health Policy.

[17] Flores G, Krishnakumar J, O’Donnell O, Van Doorslaer E (2008). Coping with health-care costs: Implications for the measurement of catastrophic expenditures and poverty. Health Econ..

[18] Garrett N, Martini EM (2007). The boomers are coming: A total cost of care model of the impact of population aging on the cost of chronic conditions in the United States. Dis. Manag..

[19] Giarda E, Moroni G (2015). `It’s a trap!’ The degree of poverty persistence in Italy and Europe.

[20] Goraus-Tańska K, Inchauste G (2016). The distributional impact of taxes and transfers in Poland.

[21] Hohberg M, Donat F, Marra G, Kneib T (2021). Beyond unidimensional poverty analysis using distributional copula models for mixed ordered-continuous outcomes. J. Roy. Stat. Soc.: Ser. C (Appl. Stat.).

[22] Hsu J, Flores G, Evans D, Mills A, Hanson K (2018). Measuring financial protection against catastrophic health expenditures: methodological challenges for global monitoring. Int. J. Equity Health.

[23] Kim E, Kwon S (2021). The effect of catastrophic health expenditure on exit from poverty among the poor in South Korea. Int. J. Health Plann. Manag..

[24] Kronenberg C, Barros PP (2014). Catastrophic healthcare expenditure - drivers and protection: the Portuguese case. Health Policy.

[25] Łuczak J, García-Gómez P (2012). Financial burden of drug expenditures in Poland. Health Policy.

[26] Marra, G., Radice, R.: (2019) GJRM: generalised joint regression modelling. R package version 0.2.

[27] Marra G, Radice R, Filippou P (2017). Regression spline bivariate probit models: a practical approach to testing for exogeneity. Commun. Stat.-Simul. Comput..

[28] Maruotti A (2009). Fairness of the national health service in Italy: a bivariate correlated random effects model. J. Appl. Stat..

[29] Mchenga M, Chirwa GC, Chiwaula LS (2017). Impoverishing effects of catastrophic health expenditures in Malawi. Int. J. Equity Health.

[30] Mendelson DN, Schwartz WB (1993). The effects of aging and population growth on health care costs. Health Aff..

[31] Meyer BD, Sullivan JX (2012). Identifying the disadvantaged: official poverty, consumption poverty, and the new supplemental poverty measure. J. Econ. Perspect..

[32] Mokrzycka A, Kowalska-Bobko I, Sagan A, Włodarczyk W (2016). The 2014 primary health care reform in Poland: short-term fixes instead of a long-term strategy. Health Policy.

[33] Mussida C, Sciulli D (2021). Childbirth and poverty in Europe: a dynamic bivariate approach. Rev. Income Wealth.

[34] OECD (2019). Society at a glance 2019: OECD social indicators.

[35] OECD/European Observatory on Health Systems and Policies (2019). Poland: country health profile 2019, state of health in the EU.

[36] OECD, European Union (2020). Health at a glance: Europe 2020: state of health in the EU cycle.

[37] OECD (2020). Waiting times for health services: next in line.

[38] Özgen Narcı H, Şahin İ, Yıldırım HH (2015). Financial catastrophe and poverty impacts of out-of-pocket health payments in Turkey. Eur. J. Health Econ..

[39] Park S, Kim B, Kim S (2016). Poverty and working status in changes of unmet health care need in old age. Health Policy.

[40] Sanwald A, Theurl E (2017). Out-of-pocket expenditures for pharmaceuticals: lessons from the Austrian household budget survey. Eur. J. Health Econ..

[41] Scheil-Adlung X, Bonan J (2013). Gaps in social protection for health care and long-term care in Europe: Are the elderly faced with financial ruin?. Int. Soc. Secur. Rev..

[42] Shorrocks AF (1978). The measurement of mobility. Econ. J. Econ. Soc..

[43] Short K (2012). The research supplemental poverty measure: 2011.

[44] Sirag A, Nor NM (2021). Out-of-pocket health expenditure and poverty: evidence from a dynamic panel threshold analysis. Healthcare.

[45] Sowada C, Sagan A, Kowalska-Bobko I (2019). Poland: a health system review.

[46] Tambor M, Pavlova M (2020). Can people afford to pay for health care? New evidence on financial protection in Poland. Summary.

[47] United Nations Department of Economic and Social Affairs (2020). World population ageing 2019.

[48] Van Doorslaer E, O’Donnell O, Rannan-Eliya RP, Somanathan A, Adhikari SR, Garg CC, Harbianto D, Herrin AN, Huq MN, Ibragimova S (2007). Catastrophic payments for health care in Asia. Health Econ..

[49] Van Doorslaer E, O’Donnell O, Rannan-Eliya RP, Somanathan A, Adhikari SR, Garg CC, Harbianto D, Herrin AN, Huq MN, Ibragimova S, Karan A, Ng CW, Pande BR, Racelis R, Tao S, Tin K, Tisayaticom K, Trisnantoro L, Vasavid C, Zhao Y (2006). Effect of payments for health care on poverty estimates in 11 countries in Asia: an analysis of household survey data. Lancet.

[50] Wagstaff A, Cotlear D, Eozenou PH-V, Buisman LR (2016). Measuring progress towards universal health coverage: with an application to 24 developing countries. Oxf. Rev. Econ. Policy.

[51] Wagstaff A, O’Donnell O, Van Doorslaer E, Lindelow M (2007). Analyzing health equity using household survey data: a guide to techniques and their implementation.

[52] World Health Organization (2020). Global monitoring report on financial protection in health 2019.

[53] Xu K, Evans DB, Carrin G, Aguilar-Rivera AM, Musgrove P, Evans T (2007). Protecting households from catastrophic health spending. Health Aff..

[54] Xu K, Evans DB, Kawabata K, Zeramdini R, Klavus J, Murray CJL (2003). Household catastrophic health expenditure: a multicountry analysis. Lancet.

[55] Yerramilli P, Fernández Ó, Thomson S (2018). Financial protection in Europe: a systematic review of the literature and mapping of data availability. Health Policy.

[56] Yildirim J, Bakır MA, Savas A (2018). State dependence in poverty: the case of Turkey. Emerg. Mark. Financ. Trade.

[57] Zawada A, Kolasa K, Kronborg C, Rabczenko D, Rybnik T, Lauridsen JT, Ceglowska U, Hermanowski T (2017). A comparison of the burden of out-of-pocket health payments in Denmark, Germany and Poland. Glob. Policy.

[58] Zhou Y, Wushouer H, Vuillermin D, Guan X, Shi L (2021). Does the universal medical insurance system reduce catastrophic health expenditure among middle-aged and elderly households in China? A longitudinal analysis. Eur. J. Health Econ..

