# Supplementary material for: The causal effect of catastrophic health expenditure on poverty in Poland
Source: Eur J Health Econ. 2023 Mar 10;25(2):193–206. doi: 10.1007/s10198-023-01579-6 (PMC9999341; doi:10.1007/s10198-023-01579-6)
Supplement: Supplementary file 1 — Supplementary file1 (DOCX 47 KB) [file 10198_2023_1579_MOESM1_ESM.docx]

**Supplementary Appendix**

Table A1. Poverty rate by age groups and poverty indicators

| Age of household head | Poor according to the CSO indicator, but not-poor according to the OPM-free indicator | Poor according to the OPM-free indicator, but not-poor according to the CSO indicator |
| --- | --- | --- |
| under 70 | 1.00% | 0.80% |
| 70 or over | 0.50% | 2.90% |

Notes: Authors' estimates based on 2018 Polish HBS data; CSO indicator: relative poverty calculated on the basis of total consumption; OPM-free indicator: relative poverty calculated on the basis of total consumption minus OPM expenses.

Table A2. Number of observations

| panel | obs. no. |
| --- | --- |
| 2010-2011 | 14,642 |
| 2011-2012 | 14,513 |
| 2012-2013 | 14,809 |
| 2016-2017 | 15,295 |
| 2017-2018 | 14,927 |

Table A3. One-dimensional mobility measures

|  | | CHE measure | Immobility ratio (Shorrocks,1978) | |
| --- | --- | --- | --- | --- |
| CHE with  threshold 25% | normative spending approach | | 0.65 [0.65,0.66] | 0.69 [0.68,0.70] |
| CHE with  threshold 30% | normative spending approach | | 0.65 [0.64,0.65] | 0.70 [0.69,0.73] |
| CHE with  threshold 35% | normative spending approach | | 0.64 [0.64,0.65] | 0.71 [0.71,0.78] |
| CHE with  threshold 40% | normative spending approach | | 0.63 [0.62,0.63] | 0.74 [0.73,0.83] |
| CHE with  threshold 10% | budget share approach | | 0.66 [0.66,0.66] | 0.68 [0.67,0.69] |
| CHE with  threshold 15% | budget share approach | | 0.63 [0.63,0.64] | 0.73 [0.72,0.74] |
| CHE with  threshold 20% | budget share approach | | 0.61 [0.60,0.62] | 0.78 [0.77,0.79] |
| CHE with  threshold 25% | budget share approach | | 0.58 [0.58,0.59] | 0.83 [0.81,0.85] |
| OPM-free poverty (households with CHE at $t-1$) | normative spending approach (threshold=40%) | | 0.71 [0.69,0.72] | 0.58 [0.56,0.62] |
| OPM-free poverty (households with CHE at $t$) | normative spending approach (threshold=40%) | | 0.75 [0.74,0.77] | 0.49 [0.46,0.52] |
| OPM-free poverty (households with CHE at $t-1$) | budget share approach (threshold=15%) | | 0.71 [0.70,0.72] | 0.58 [0.56,0.61] |
| OPM-free poverty (households with CHE at $t$) | budget share approach (threshold=15%) | | 0.75 [0.74,0.77] | 0.49 [0.46,0.52] |
| OPM free poverty |  | | 0.73 [0.73,0.73] | 0.54 [0.53,0.55] |
| CSO poverty |  | | 0.73 [0.73,0.74] | 0.53 [0.52,0.54] |

Notes: Authors' estimates based on the Polish HBS data; 95% bootstrap intervals in brackets; CSO poverty: relative poverty calculated on the basis of total consumption; OPM free poverty: relative poverty calculated on the basis of total consumption minus OPM expenses; the immobility ratio: the sum of diagonal elements of a transition matrix, Shorrocks (1978) mobility index: one minus the second greatest eigenvalue of a transition matrix.

| Table A4. Transition matrices for the incidence of CHE | budget share approach | threshold=15% | without CHE (t) | 67.1 | 94 |  | 91.7 | threshold=25% | without CHE (t) | 81.4 | 98.2 | 97.8 | normative spending approach | threshold=30% | without CHE (t) | 62.7 | 93.4 | 90.4 | threshold=40% | without CHE (t) | 70.0 | 96.4 | 95.1 | Notes: Authors' estimates based on the Polish HBS data, number of observations 74,186. |
| --- | --- | --- | --- | --- | --- | --- | --- | --- | --- | --- | --- | --- | --- | --- | --- | --- | --- | --- | --- | --- | --- | --- | --- | --- |
|  |  |  | with CHE (t) | 32.9 | 6.0 |  | 8.3 |  | with CHE (t) | 18.6 | 1.8 | 2.2 |  |  | with CHE (t) | 37.3 | 6.6 | 9.6 |  | with CHE (t) | 30.0 | 3.6 | 4.9 |  |
|  |  |  |  | With CHE (t-1) | Without CHE (t-1) |  | shares at time t |  |  | With CHE (t-1) | Without CHE (t-1) | shares at time t |  |  |  | with CHE (t-1) | without CHE (t-1) | shares at time t |  |  | with CHE (t-1) | without CHE (t-1) | shares at time t |  |
|  |  | threshold=10% | without CHE (t) | 56.9 | 88.5 |  | 82.8 | threshold=20% | without CHE (t) | 74.7 | 96.8 | 95.8 |  | threshold=25% | without CHE (t) | 59.3 | 91.1 | 86.7 | threshold=35% | without CHE (t) | 66.2 | 95.2 | 93.1 |  |
|  |  |  | with CHE (t) | 43.1 | 11.5 |  | 17.2 |  | with CHE (t) | 25.3 | 3.2 | 4.2 |  |  | with CHE (t) | 40.7 | 8.9 | 13.3 |  | with CHE (t) | 33.8 | 4.8 | 6.9 |  |
|  |  |  |  | with CHE (t-1) | Without CHE (t-1) |  | shares at time t |  |  | With CHE (t-1) | Without CHE (t-1) | shares at time t |  |  |  | with CHE (t-1) | without CHE (t-1) | shares at time t |  |  | with CHE (t-1) | without CHE (t-1) | shares at time t |  |

Table A5. Definitions of variables

| Variable | Definition |
| --- | --- |
| $POV$ | binary variable, takes one if a household is currently in OPM-free poverty |
| $CHE$ | binary variable, takes one if a household currently has CHE |
| $POV_{-1}$ | binary variable, takes one if a household was in OPM-free poverty in the previous year |
| $CHE_{-1}$ | binary variable, takes one if a household had CHE in the previous year |
| HH age<35 and >24 | binary variable, takes one if age of a household is between 25 and 34 |
| HH age<45 and >34 | binary variable, takes one if age of a household is between 35 and 44 |
| HH age<55 and >44 | binary variable, takes one if age of a household is between 45 and 54 |
| HH age<65 and >54 | binary variable, takes one if age of a household is between 55 and 64 |
| HH age<75 and >64 | binary variable, takes one if age of a household is between 65 and 74 |
| HH age>74 | binary variable, takes one if age of a household greater than 74 |
|  | baseline: age of a household is lower than 25 |
| Disabled in household | binary variable, takes one if there is a disabled person in a household |
| No. of children | the number of children in a household |
| No. of working | the number of working adults in a household |
| No. of not working | the number of not working adults in a household |
| HH is a male | binary variable, takes one if a household head is a male |
| HH is in a relationship | binary variable, takes one if a household head is in a relationship |
| HH has an academic degree | binary variable, takes one if a household head has an academic degree |
| Income from farming | binary variable, takes one if a household's major source of income comes from farming |
| Income from self-emp. | binary variable, takes one if a household's major source of income comes from self-employment |
| Lives in a town | binary variable, takes one if a household lives in a town |
| Lives in a village | binary variable, takes one if a household lives in a village |
| Region1 | binary variable, takes one if a household lives in the central region of Poland |
| Region2 | binary variable, takes one if a household lives in the south region of Poland |
| Region3 | binary variable, takes one if a household lives in the east region of Poland |
| Region4 | binary variable, takes one if a household lives in the north-west region of Poland |
| Region5 | binary variable, takes one if a household lives in the south-west region of Poland |
|  | baseline: north region of Poland |
| Year11 | binary variable, takes one if time t equals 2011 |
| Year12 | binary variable, takes one if time t equals 2012 |
| Year13 | binary variable, takes one if time t equals 2013 |
| Year17 | binary variable, takes one if time t equals 2017 |
|  | baseline: time t equals 2018 |

Table A6. Descriptive statistics of continuous/categorical variables

| Variable | Mean | St. dev. |
| --- | --- | --- |
| No. of children | 1.12 | 0.99 |
| No. of working | 1.8 | 1.05 |
| No. of not working | 0.58 | 0.94 |

Table A7. Ratios for dummy variables

| Variable | Ratio |
| --- | --- |
| HH age<35 and >24 | 0.12 |
| HH age<45 and >34 | 0.18 |
| HH age<55 and >44 | 0.2 |
| HH age<65 and >54 | 0.23 |
| HH age<75 and >64 | 0.15 |
| HH age>74 | 0.1 |
| HH is a male | 0.62 |
| HH is in a relationship | 0.67 |
| HH has an academic degree | 0.2 |
| Income from farming | 0.05 |
| Income from self-emp. | 0.07 |
| Lives in a town | 0.11 |
| Lives in a village | 0.45 |
| Disabled in household | 0.21 |
| Region1 | 0.22 |
| Region2 | 0.2 |
| Region3 | 0.18 |
| Region4 | 0.15 |
| Region5 | 0.1 |
| Year11 | 0.2 |
| Year12 | 0.2 |
| Year13 | 0.2 |
| Year17 | 0.21 |

Table A8. State dependence and feedback effects, poverty and CHE, poverty cutoff=0.45

| budget share approach | | | | | | | | |
| --- | --- | --- | --- | --- | --- | --- | --- | --- |
| threshold | 10% | | 15% | | 20% | | 25% | |
|  | poverty equation | | | | | | | |
|  | AME | s.e. | AME | s.e. | AME | s.e. | AME | s.e. |
|  |  |  |  |  |  |  |  |  |
| 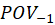 | 0.300 *** | 0.127 | 0.296 *** | 0.124 | 0.295 *** | 0.121 | 0.295 *** | 0.119 |
| 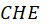 | 0.125 *** | 0.079 | 0.173 *** | 0.094 | 0.195 *** | 0.098 | 0.237 *** | 0.108 |
| 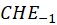 | -0.028*** | 0.027 | -0.025 *** | 0.025 | -0.022 *** | 0.022 | -0.018 * | 0.017 |
|  | CHE equation | | | | | | | |
|  | AME | s.e. | AME | s.e. | AME | s.e. | AME | s.e. |
| 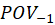 | -0.02 *** | 0.01 | -0.005 | 0.004 | 0.001 | 0.001 | 0.001 | 0.001 |
| 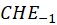 | 0.195 *** | 0.055 | 0.150 *** | 0.066 | 0.112 *** | 0.064 | 0.081 *** | 0.053 |
| normative spending approach | | | | | | | | |
| threshold | 25% | | 30% | | 35% | | 40% | |
|  | poverty equation | | | | | | | |
|  | AME | s.e. | AME | s.e. | AME | s.e. | AME | s.e. |
| 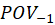 | 0.277 *** | 0.125 | 0.282 *** | 0.123 | 0.285 *** | 0.12 | 0.286 *** | 0.119 |
| 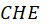 | 0.148 *** | 0.089 | 0.12 *** | 0.076 | 0.09 *** | 0.061 | 0.082 ** | 0.056 |
| 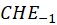 | -0.011 ** | 0.011 | -0.007 | 0.006 | -0.001 | 0.001 | 0.004 | 0.003 |
|  | CHE equation | | | | | | | |
|  | AME | s.e. | AME | s.e. | AME | s.e. | AME | s.e. |
| 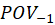 | 0.033 *** | 0.021 | 0.026 *** | 0.018 | 0.028 *** | 0.023 | 0.027 *** | 0.024 |
| 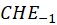 | 0.160 *** | 0.069 | 0.156 *** | 0.074 | 0.139 *** | 0.077 | 0.119 *** | 0.075 |

Notes: Authors' estimates based on the recursive bivariate probit models and the Polish HBS panel; AME expresses the average marginal effect of the change from 0 to 1; ***, **, *, and . denote parameter significance at the 0.1%, 1%, 5% and 10% levels, respectively.

Table A9. The causal impact of a new incidence of CHE on relative poverty, poverty cutoff=0.45

| budget share approach | | | | |
| --- | --- | --- | --- | --- |
| threshold | 10% | 15% | 20% | 25% |
| risk ratio | 2.69 [2.00, 3.38] | 3.2 [2.30, 4.08] | 3.38 [2.08, 4.71] | 3.82 [1.97, 5.77] |
| odds ratio | 3.11 [2.18, 4.15] | 3.94 [2.60, 5.48] | 4.29 [2.31, 6.98] | 5.15 [2.17, 10.19] |
|  |  |  |  |  |
| normative spending approach | | | | |
| threshold | 25% | 30% | 35% | 40% |
| risk ratio | 3.08 [2.28, 3.88] | 2.59 [1.85, 3.35] | 2.14 [1.36, 3.01] | 2.01 [1.16, 2.99] |
| odds ratio | 3.67 [2.54, 4.93] | 2.98 [2.00, 4.10] | 2.37 [1.40, 3.59] | 2.20 [1.18, 3.59] |

Notes: Authors' estimates based on the recursive bivariate models and the Polish HBS panel; 95% confidence intervals are based on posterior simulations.

Table A10. State dependence and feedback effects, poverty and CHE, poverty cutoff=0.55

| budget share approach | | | | | | | | |
| --- | --- | --- | --- | --- | --- | --- | --- | --- |
| threshold | 10% | | 15% | | 20% | | 25% | |
|  | poverty equation | | | | | | | |
|  | AME | s.e. | AME | s.e. | AME | s.e. | AME | s.e. |
| 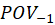 | 0.354 *** | 0.109 | 0.354 *** | 0.107 | 0.351 *** | 0.105 | 0.350 *** | 0.104 |
| 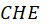 | 0.181 *** | 0.08 | 0.197 *** | 0.08 | 0.320 *** | 0.101 | 0.433 *** | 0.115 |
| 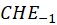 | -0.048 *** | 0.033 | -0.041 *** | 0.029 | -0.046 *** | 0.034 | -0.048 *** | 0.035 |
|  | CHE equation | | | | | | | |
|  | AME | s.e. | AME | s.e. | AME | s.e. | AME | s.e. |
| 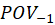 | -0.019 *** | 0.009 | -0.005 | 0.004 | 0.000 | 0.000 | 0.002 | 0.002 |
| 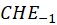 | 0.195 *** | 0.055 | 0.150 *** | 0.066 | 0.112 *** | 0.064 | 0.080 *** | 0.052 |
| normative spending approach | | | | | | | | |
| threshold | 25% | | 30% | | 35% | | 40% | |
|  | poverty equation | | | | | | | |
|  | AME | s.e. | AME | s.e. | AME | s.e. | AME | s.e. |
| 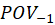 | 0.334 *** | 0.109 | 0.339 *** | 0.108 | 0.344 *** | 0.106 | 0.345 *** | 0.105 |
| 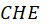 | 0.281 *** | 0.102 | 0.236 *** | 0.091 | 0.213 *** | 0.084 | 0.244 *** | 0.089 |
| 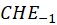 | -0.034 *** | 0.026 | -0.024 *** | 0.017 | -0.021 ** | 0.015 | -0.026 *** | 0.018 |
|  | CHE equation | | | | | | | |
|  | AME | s.e. | AME | s.e. | AME | s.e. | AME | s.e. |
| 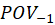 | 0.030 *** | 0.019 | 0.023 *** | 0.016 | 0.024 *** | 0.02 | 0.022 *** | 0.021 |
| 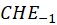 | 0.154 *** | 0.067 | 0.151 *** | 0.073 | 0.135 *** | 0.076 | 0.116 *** | 0.074 |

Notes: Authors' estimates based on the recursive bivariate probit models and the Polish HBS panel; AME expresses the average marginal effect of the change from 0 to 1; ***, **, *, and . denote parameter significance at the 0.1%, 1%, 5% and 10% levels, respectively.

Table A11. The causal impact of a new incidence of CHE on relative poverty, poverty cutoff=0.55

| budget share approach | | | | |
| --- | --- | --- | --- | --- |
| threshold | 10% | 15% | 20% | 25% |
| risk ratio | 2.22 [1.81, 2.60] | 2.24 [1.74, 2.69] | 2.97 [2.35, 3.51] | 3.62 [3.00, 4.08] |
| odds ratio | 2.82 [2.13, 3.56] | 2.93 [2.04, 3.92] | 4.81 [3.19, 6.74] | 7.53 [4.97, 10.54] |
|  |  |  |  |  |
| normative spending approach | | | | |
| threshold | 25% | 30% | 35% | 40% |
| risk ratio | 2.97 [2.48, 3.41] | 2.58 [2.11, 2.99] | 2.37 [1.86, 2.83] | 2.55 [2.09, 2.94] |
| odds ratio | 4.42 [3.32, 5.58] | 3.57 [2.64, 4.55] | 3.17 [2.22, 4.2] | 3.58 [2.64, 4.59] |

Notes: Authors' estimates based on the recursive bivariate models and the Polish HBS panel; 95% confidence intervals are based on posterior simulations.

Table A12. Poverty equation, AME of control variables

|  | budget share approach | | | | | | | |
| --- | --- | --- | --- | --- | --- | --- | --- | --- |
| threshold | 10% |  | 15% |  | 20% |  | 25% |  |
|  | AME | s.e. | AME | s.e. | AME | s.e. | AME | s.e. |
| HH age<35 and >24 | 0.025 * | 0.018 | 0.025 * | 0.018 | 0.026 * | 0.019 | 0.026 * | 0.019 |
| HH age<45 and >34 | 0.022 . | 0.016 | 0.023 * | 0.016 | 0.023 * | 0.017 | 0.024 * | 0.017 |
| HH age<55 and >44 | 0.028 * | 0.020 | 0.029 * | 0.021 | 0.029 ** | 0.021 | 0.03 ** | 0.021 |
| HH age<65 and >54 | 0.019 . | 0.014 | 0.021 . | 0.015 | 0.022 * | 0.017 | 0.023 * | 0.017 |
| HH age<75 and >64 | 0.000 | 0.000 | 0.004 | 0.003 | 0.007 | 0.005 | 0.009 | 0.007 |
| HH age>74 | 0.016 | 0.012 | 0.021 . | 0.015 | 0.022 . | 0.016 | 0.003 * | 0.021 |
| HH is a male | 0.000 | 0.000 | -0.001 | 0.001 | -0.001 | 0.001 | -0.001 | 0.001 |
| HH is in a relationship | -0.029 *** | 0.022 | -0.028 *** | 0.021 | -0.028 *** | 0.021 | -0.027 *** | 0.020 |
| HH has academic degree | -0.076 *** | 0.065 | -0.075 *** | 0.065 | -0.074 *** | 0.066 | -0.074 *** | 0.065 |
| Income from farming | 0.02 *** | 0.014 | 0.02 *** | 0.015 | 0.02 *** | 0.015 | 0.021 *** | 0.015 |
| Income from self-emp. | -0.019 *** | 0.015 | -0.02 *** | 0.016 | -0.02 *** | 0.016 | -0.02 *** | 0.016 |
| Lives in a town | -0.036 *** | 0.030 | -0.036 *** | 0.030 | -0.036 *** | 0.031 | -0.036 *** | 0.030 |
| Lives in a village | 0.037 *** | 0.027 | 0.036 *** | 0.027 | 0.036 *** | 0.027 | 0.036 *** | 0.027 |
| Disabled in household | 0.002 | 0.002 | 0.003 | 0.002 | 0.003 | 0.002 | 0.004 | 0.003 |
| Nb of working | -0.018 *** | 0.121 | -0.019 *** | 0.124 | -0.02 *** | 0.126 | -0.02 *** | 0.127 |
| Nb of not working | 0.038 *** | 0.156 | 0.038 *** | 0.156 | 0.038 *** | 0.157 | 0.038 *** | 0.155 |
| Nb of children | 0.033 *** | 0.151 | 0.033 *** | 0.149 | 0.032 *** | 0.148 | 0.032 *** | 0.146 |
